# Evaluating the Impact of Community-Based Medical Education on Health Literacy and Patient Empowerment in Underserved Populations: A Pilot Cohort Study

**DOI:** 10.3390/clinpract15060097

**Published:** 2025-05-22

**Authors:** Aida Aljafri, Persia Abba, Anita Sedghi, Andreas Conte, Waseem Jerjes

**Affiliations:** 1 Brighton & Sussex Medical School, Brighton BN1 9PX, UK; a.aljafri1@uni.bsms.ac.uk (A.A.); p.abba1@uni.bsms.ac.uk (P.A.); 2 Faculty of Medicine, Imperial College London, London SW7 2AZ, UK; anita.sedghi@nhs.net; 3 Faculty of Life Sciences & Medicine, King’s College London, London SE1 1UL, UK; andreas.conte@kcl.ac.uk

**Keywords:** community-based education, health literacy, medical students, underserved populations, patient empowerment, reflective learning

## Abstract

**Background:** Traditionally, community-based education (CBE) programmes have been utilised for teaching medical students clinical and interpersonal skills through placement in underserved environments. This pilot cohort study tested an extended model of CBE by infusing patient education into student-conducted consultations with the dual objectives of stimulating improved learning for the students and improved health literacy for the patients. **Methods:** The intervention involved 38 final-year medical students and 85 adult patients and from underprivileged communities in North West London. The students first undertook online preparatory workshops on health literacy, communication skills, and cultural competence. Subsequently, they imparted 20–30 min educational sessions on chronic disease management and preventive care to the patients on their clinical placements. The quantitative measurement used pre- and post-intervention questionnaires, and the qualitative measurement was based on reflective diaries and patient feedback. Paired *t*-tests were used for statistical comparisons, while a thematic analysis was used for textual answers. **Results:** Student confidence in breaking down medical jargon improved from 2.8 ± 0.7 to 4.4 ± 0.5 (*p* < 0.01), and confidence in making use of visual aids improved from 2.5 ± 0.8 to 4.2 ± 0.6 (*p* < 0.01). Understanding among the patients of their health conditions improved from 27% to 74% (*p* < 0.001), and self-confidence in their ability to manage their health improved from 31% to 79% (*p* < 0.001). The qualitative feedback noted improved empathy, cultural sensitivity, and a positive effect on patient empowerment through tailored education. **Conclusions:** This CBE intervention had two benefits: improving teaching and communication skills in students and greatly enhancing health literacy in underserved patients. The integration of structured education into usual care encounters holds the promise of a scalable, sustainable method for addressing health disparities. Longer longitudinal studies are necessary to assess its long-term success and incorporation into medical education.

## 1. Background and Rationale

Community-based education (CBE) has become a cornerstone of health professionals’ education, providing students with an opportunity for clinical experience in under-resourced environments and instilling social responsibility in the healthcare profession. Historically, such programmes have focused on strengthening the clinical skills of students and their education on healthcare disparities faced by marginalised populations [1,2]. Yet, despite its value, we argue that CBE is underutilised when tackling the other pervasive health inequity: poor health literacy.

Health literacy, understood as one’s capacity to access, comprehend, and apply health information for decision-making, is an accepted health outcome determinant [3]. Low health literacy has been linked to poorer chronic disease self-management, delayed medical care, and increased hospitalisation [4,5]. For most underserved groups—defined in this context as populations experiencing socioeconomic disadvantage, linguistic barriers, or limited access to healthcare—such deficits exacerbate health outcome disparities [6]. These populations often face systemic barriers that hinder their ability to make informed decisions or engage fully with health services.

Redesigning the objectives of CBE so as to include deliberate health literacy improvement for patients provides a two-way benefit. Students not only enhance their teaching and communication skills, but patients gain improved control and understanding of their health as well [7]. There have been a number of initiatives in recent times indicating that incorporating patient education into normal student-conducted consultations enhances the empowerment of patients with an enriching experience for the training of the students [8,9].

Alongside improving health literacy, the intervention also aimed to promote patient empowerment, defined as the process through which individuals gain greater control over decisions and actions affecting their health. CBE models that embed structured education into clinical encounters provide opportunities for patients to ask questions, express concerns, and co-construct health goals with student clinicians. By encouraging dialogue and providing personalised, understandable information, the intervention sought to increase patients’ sense of agency, confidence, and participation in their care.

The sustainability of such interventions remains a major issue. Health education during placements usually ceases with the rotation, restricting its long-term influence [10]. Continuity could be ensured through planned cooperation with local healthcare providers and community groups [11], through which subsequent groups of trainee doctors could leverage the work that has gone before and ensure that health education is integrated within community health on a sustainable basis [12].

### Objectives

This pilot cohort study aimed to evaluate a novel CBE programme that integrates structured patient education into the clinical practice of final-year medical students in underserved communities. The dual objectives were:

1. To assess the impact of this programme on the students’ confidence and competence in delivering health education;
2. To examine whether the intervention improved the patients’ health literacy, confidence in self-management, and engagement in preventive practices.

We hypothesised that participation in the programme would result in measurable improvements in both student communication skills and patient-reported outcomes related to health understanding and self-care.

## 2. Methods

### 2.1. Study Design

This was a pilot cohort study with a prospective design that was aimed at assessing the effects of a systematic CBE programme on medical student competencies as well as on patient health literacy. It had a pre–post design with no control group and spanned over eight weeks. Students went through preparatory training and followed up with educational interventions in the course of usual clinic encounters with patients from underprivileged communities. It used pre- and post-intervention questionnaires and qualitative feedback instruments for measurement of outcomes (Appendix A). This study design facilitated the collection of both qualitative and quantitative data in an assessment of changes over time among the same participants (Table 1).

### 2.2. Setting

The research was carried out in community health clinics in North West London, located in boroughs identified by the UK Index of Multiple Deprivation and local public health data as areas of significant socioeconomic disadvantage. These communities have documented high rates of chronic disease, low levels of health literacy, and reduced access to preventive care services. The clinics involved also have an established track record in hosting student placements and conducting outreach to underserved groups, making them appropriate and feasible settings for the intervention [13,14].

The intervention spanned eight weeks. All educational sessions with students were integrated within normal clinical encounters alongside regular patient visits. Student training workshops were conducted remotely using online platforms, whereas patient interactions were conducted face-to-face within participating communities.

### 2.3. Participants

The participants consisted of two groups: final-year medical students and adult patients from deprived communities in North West London.

A total of 38 final-year medical students were recruited. All were invited through institutional mailing lists and participated on a voluntary basis. The inclusion criterion was that they should have had past experience of clinical or community placements.

Eighty-five adult patients were enrolled from participating community clinics on routine healthcare visits. Eligible patients were 18 years old and older, had a minimum of one chronic illness, and had limited health literacy. Limited health literacy was clinician-judged during routine consultations using informal indicators such as difficulty understanding health instructions, limited engagement with written materials, or frequent misinterpretation of clinical advice. This approach reflects established practice in primary care when formal literacy tools are not feasible [15,16]. Patients were invited by clinical staff and asked if they were willing to participate in education sessions led by medical students.

As this was an educational assessment with a service focus, there were no matching procedures or randomisation.

Demographic data collected for patients included age, gender, ethnicity, education level, and primary language. These variables were used for subgroup analysis to explore differential impacts of the intervention.

### 2.4. Variables

This research tested a variety of outcome measures for both patients and students. For medical students, these included their self-assessment of their ability to simplify medical ideas, their skill with visual aids and teach-back, their cultural sensitivity when talking with patients, and their skill in involving patients in substantive health conversations. These were assessed on a five-point Likert scale via pre- and post-intervention questionnaires, supervisor feedback, and reflective journaling.

For patients, the most important variables were their self-reported knowledge of their medical conditions, their belief that they could manage their condition, and their satisfaction with the education sessions. Patient knowledge and self-efficacy were measured using standardised before-and-after questionnaires, and session satisfaction by rating scales and qualitative feedback. Secondary variables were changes in behavioural intentions such as commitment towards preventive behaviours and greater uptake of local health services. Patient perceptions of how clear, cultural, and interpersonal the sessions were also examined using open-ended survey responses.

Diagnostic criteria were not used since educational outcomes were of major interest over clinician diagnoses.

### 2.5. Data Sources and Measurement

Data were gathered through both qualitative reflection and third-party feedback, as well as a mixture of quantitative surveys.

Data were gathered through student reflective journals and structured feedback from supervisors and patients. Reflective journals were completed throughout the placement and served as a qualitative data source capturing students’ experiences, challenges, and learning insights. These narratives were analysed thematically using Braun and Clarke’s six-phase framework for thematic analysis [17]. Reflective practice was incorporated to encourage critical thinking, empathy, and integration of clinical and communication skills [18].

Sources of data for medical students were pre- and post-programme self-completion questionnaires that were intended to measure changes in confidence, communication skills, and cultural sensitivity. Five-point Likert scales were used for these questionnaires, which were tailored specifically for the programme using tested educational evaluation instruments. Students maintained reflective journals during the placement that presented qualitative accounts of their experience and their development as health educators. Student performance was evaluated by supervisors through observation of them in action during clinic sessions, observing the clear communication of ideas, patient engagement, and application of health literacy concepts.

For the patients, measurement occurred through structured pre- and post-session questionnaires that were completed both before and after the educational interactions with the students. These consisted of closed-ended items measuring understanding, self-management confidence, and satisfaction, as well as open-ended questions for qualitative feedback. The questionnaires were written using plain language and visual aids where necessary, making them suitable for patients with low literacy.

Moreover, multidisciplinary healthcare professionals from participating clinics offered feedback through brief structured interviews or written feedback. Their opinions were utilised in the evaluation of feasibility, context relevance, and impact of the intervention on daily care. All methods of data collection were applied equally across participants, and there were no variations in the approach to assessment between groups.

### 2.6. Bias

A number of steps were used to reduce possible sources of bias in the present study. Selection bias was limited by inviting all the final-year medical students from participating institutions and recruiting all participants with predefined inclusion criteria during their regular clinical interactions. Participation was voluntary for both groups, and informed consent was sought for ethical transparency.

Response bias was eliminated through anonymising both survey answers and reflective diaries, promoting frank and unfettered feedback. Observer bias was minimised by using other healthcare professionals in assessing the performance of the students, and uniform rating scales were used.

Students’ and patients’ answers could have been affected by social desirability bias, particularly on self-reporting questionnaires. To alleviate that, questionnaires contained both positive- and neutral-framed items, and qualitative feedback was cross-checked with qualitative measures to verify findings. Pre- and post-intervention designs served to limit recall bias because measures were made close to the sessions.

Due to the pilot nature of this educational study, participant and assessor blinding was not possible. Despite that, reflective journaling and thematic analysis enabled richer understanding of the patient and student experiences, with contextual depth and credibility added by the reported outcomes.

### 2.7. Thematic Analysis of Reflective Journals

Reflective journals completed by medical students during the placement were analysed using thematic analysis following the six-phase framework described by Braun and Clarke [17,19]. Journals were read in full by two researchers, who independently generated initial codes using an inductive approach. Coding was conducted manually using NVivo (version 14) software, and a coding framework was developed collaboratively through discussion. Themes were then identified, reviewed, and refined through iterative dialogue until consensus was reached. To enhance rigour, triangulation was achieved by comparing findings with supervisor and patient feedback, and an audit trail was maintained throughout the process. Reflexivity was also applied by the researchers to consider their positionality and prior assumptions when interpreting the data. Final themes were discussed with a third team member to validate coherence and relevance to the study objectives.

### 2.8. Study Size

The study size was determined in advance to ensure sufficient power to detect clinically meaningful improvements in patient health literacy. Drawing on effect sizes reported in similar health education interventions, an absolute increase of 40–50% in patient understanding was expected. Using an alpha of 0.05 and a power of 80%, the estimated number needed to treat (NNT) was approximately 2.5, meaning that for every 2 to 3 patients receiving the intervention, one would experience a significant improvement in health literacy. To detect this change with statistical confidence, a minimum of 80 patients was required. The final sample of 85 patients exceeded this threshold.

The inclusion of 38 final-year medical students was based on the full cohort available during the study period and was considered sufficient to deliver the intervention, allow repeated patient interactions, and ensure diversity in educational experience. Each student conducted educational sessions with 2 to 4 different patients, providing adequate data for within-subject comparisons and supporting the qualitative evaluation of learning outcomes. While not powered for detecting small differences between subgroups of students, this number was appropriate for a pilot evaluation focused on feasibility, acceptability, and thematic saturation in reflective analysis.

### 2.9. Quantitative Variables

Quantitative variables for the study came from both structured pre- and post-intervention questionnaires completed by patients and students. Among medical students, continuous variables consisted of Likert scale ratings from 1 through 5 of their confidence in simplifying medical information, employing visual aids, involving patients in conversation, and using culturally sensitive communication. The ratings were scored on an interval level for the purpose of statistical analysis. Categorical variables were supervisor-rated communication competence on a rating of “not competent”, “developing”, “competent”, or “highly competent”.

For patients, the key quantitative variables were binary and category measures of self-efficacy and health literacy. These were measured by patient reporting on understanding of their condition (yes/no), confidence in self-managing their condition (5-point scale), and preventive health behavioural intentions (e.g., willingness to observe for symptoms, adherence with medication). Session satisfaction was rated on a 4-point scale from “not helpful” to “very helpful”. Pre- and post-session comparisons enabled calculation of absolute change on these measures.

Continuous variables were expressed with means and standard deviations, and categorical variables were presented as percentages. The same response instruments and scales were utilised before and after intervention for comparability, as well as in an attempt to minimise measurement bias.

### 2.10. Statistical Methods

Descriptive statistics were utilised for summarising participant demographics and baseline variables. Means and standard deviations were calculated on continuous variables from Likert scale responses. For category outcomes, proportions and changes were presented as percentages. For evaluation of change in student and patient outcomes before and after intervention, paired sample *t*-tests were used for continuous variables that followed a normal distribution, and for paired category data, McNemar’s test was utilised.

No subgroup and interaction analyses were performed, because the study was not powered for stratified comparisons. Missing values were few and were not imputed; complete cases only were entered into the analytic model. All of the analyses were two-sided, with *p* < 0.05 used as a criterion for significance. SPSS version 28.0 (IBM Corporation) was used for analyses.

Since this was a pilot study, no sensitivity analyses were anticipated. The attention was on detecting significant within-subject change and on gauging feasibility, not on making generalisable and adjusted estimates.

### 2.11. Ethical Considerations

This study was reviewed using the UK Health Research Authority (HRA) decision tool, which confirmed that NHS Research Ethics Committee (REC) review was not required, as the project did not involve NHS services or the use of identifiable patient data. All participants provided informed consent, and the study was conducted in accordance with institutional ethical guidelines for educational research. A copy of the HRA decision outcome is included in the Supplementary Materials.

Participation in the research was voluntary, with all the students having consented in writing before being accepted into the programme. Patients who gave consent to participate also consented after being fully briefed regarding the objectives of the research and the procedures, as well as the non-intrusive role of the student. For maintaining confidentiality, all the data were anonymised, with the participants assigned unique study identifiers with no personally identifiable information gathered. Responses from the surveys as well as the reflection journals were stored as per the policy of data protection.

This study was conducted in accordance with the Declaration of Helsinki, ensuring that all participants were fully informed about the nature of the study, their voluntary participation, and their right to withdraw at any time without consequence.

## 3. Results

### 3.1. Participants

All 38 last-year medical students who entered the programme completed the full intervention and assessment elements. The cohort consisted of 55% females and 45% males with a mean age of 24.9 years (range: 23–28). Seventy-four percent of the students were multilingual, and 68% had prior informal experience in teaching or health education.

There were 85 patients that attended the educational sessions, of which 81 (95.3%) filled in the pre- and post-session questionnaires. There were no withdrawn patients and/or medical students, and there were no missing key outcome measure data. The patient population was 59% females and 41% males with a mean age of 53.2 years (range: 21–84). Seventy-eight percent were from a minority ethnic group and 51% indicated that their first language was not English. All of the patients provided written informed consent before participation.

Two to four patients were seen by each of the medical students during the eight-week placement, giving a total of 132 individual educational encounters between medical students and patients.

### 3.2. Baseline Characteristics and Contextual Findings

The baseline measures showed a moderate readiness among the students for patient education delivery. The confidence in explaining medical concepts was scored with a mean of 2.8 ± 0.7, followed by visual aids and teach-back with scores of 2.5 ± 0.8. Health literacy concepts were understood by 3.2 ± 0.6 and cultural competence by 3.1 ± 0.7 (Table 2). Only 40% of the students indicated confidence in starting patient conversations, and supervisors scored 42% of the students as “competent” and “highly competent” communicators prior to the programme.

The patients had a considerable burden of low health literacy. Only 27% had an understanding of their condition that enabled them to manage it, while 31% of them were confident in their self-management abilities (Table 3). Preventive practices were followed by 48%, and only 32% had knowledge of community health services. Only 45% of them felt they were engaged in their usual clinical visits, highlighting the importance of an education intervention.

### 3.3. Outcome Data

**Figure 1:**
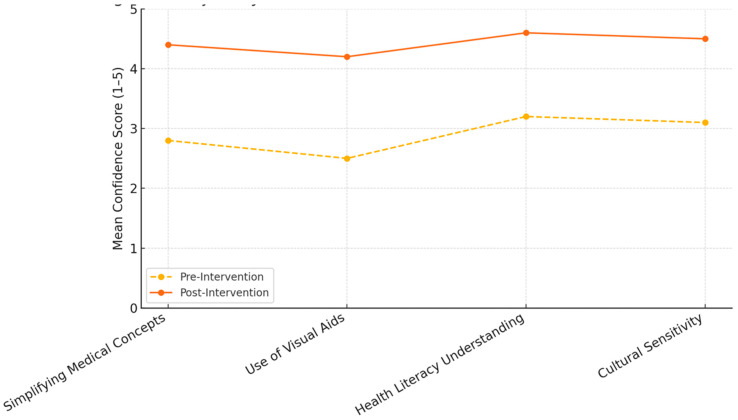
Trajectory of student confidence across core communication domains. This figure illustrates the change in mean student confidence scores (rated on a 5-point scale) across four communication domains before and after participating in the CBE programme. Confidence in simplifying medical concepts rose from 2.8 to 4.4, in the use of visual aids from 2.5 to 4.2, in understanding health literacy from 3.2 to 4.6, and in cultural sensitivity from 3.1 to 4.5. The consistent upward trajectory across all domains reflects the broad-based impact of the intervention on student development.

The results after the intervention indicated improved outcomes for all aspects of the students (Figure 1). The average confidence in simplifying medical concepts improved to 4.4 ± 0.5, in using visual aids and teach-back to 4.2 ± 0.6 (each *p* < 0.01), and in applying health literacy concepts to 4.6 ± 0.3 (*p* < 0.001). Cultural sensitivity also improved, with 87% of the students feeling more confident in cross-cultural communication. The supervisor ratings of 85% of the students stated they were “competent” or “highly competent” after the intervention (Table 2). The students identifying education as essential increased to 92% (from 54%) and confidence in managing patient expectations increased to 81% (from 37%).

Among the patients, knowledge of their condition improved from 27% to 74% (*p* < 0.001), and confidence in managing their health improved from 31% to 79% (*p* < 0.001). Attendance in education sessions was 89%, with 87% stating the information was clear. Ninety-one percent of them scored the sessions as “helpful” or “very helpful”. There was an improvement in willingness to take preventive behaviours from 48% to 82%, and 78% said they became aware of health services (Table 3). The patient’s awareness of community health resources increased by 46%.

### 3.4. Main Results

**Figure 2:**
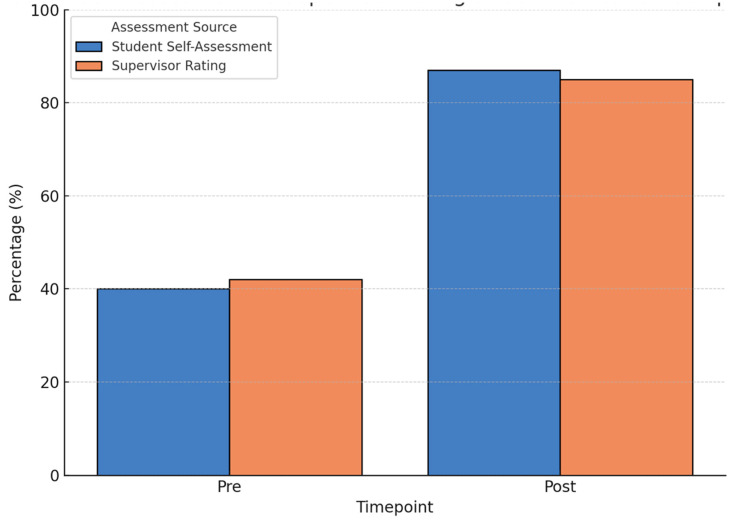
Student self-assessment vs. supervisor rating of communication competence (pre- and post-intervention). This figure compares four categories: Student self-assessment (Pre-intervention), Student self-assessment (Post-intervention), Supervisor rating (Pre-intervention), and Supervisor rating (Post-intervention). At the start of the programme, 40% of students reported confidence in engaging patients (Student self-assessment, Pre), while 42% were rated as competent or highly competent by supervisors (Supervisor rating, Pre). After the programme, these figures rose to 87% and 85%, respectively (Student self-assessment and Supervisor rating, Post). The close alignment between student and supervisor evaluations after the intervention suggests improved communication competence and self-awareness.

The effect size analysis also ensured that the educational effect was statistically significant and of considerable size (Figure 2). For the students, the Cohen’s d for simplifying medical concepts was 3.30, for visual aids and teach-back 2.13, and for understanding health literacy concepts 2.75—all of which indicated very large effect sizes.

**Figure 3:**
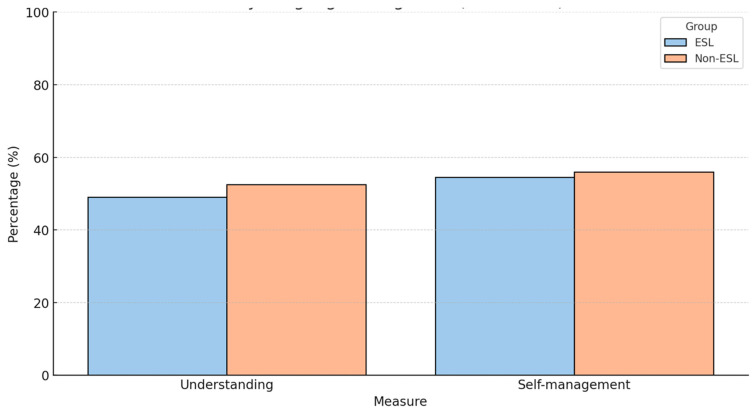
Improvement in patient understanding and self-management by language background (pre- and post-intervention). This figure illustrates the percentage of patients reporting adequate understanding of their health condition and confidence in self-management before and after participation in the CBE intervention. Data are stratified by language background: patients with English as a second language (ESL) and those with English as their first language (Non-ESL). ESL patients showed greater relative improvements in both domains, particularly in health understanding (22% to 76%), compared to Non-ESL patients (34% to 71%). These findings highlight the intervention’s potential to address disparities in health literacy among linguistically diverse populations.

The stratified exploratory analyses indicated that the students with experience in informal teaching reported higher post-intervention confidence in explaining things in simple terms, and the multilingual students improved more in terms of cultural sensitivity. For the patients, understanding was the most improved (progressing from 22% to 76%), as well as confidence in their ability to manage their conditions (progressing from 28% to 81%) (Figure 3).

These results further validate the intervention strength and relevance, with sustained progress through both quantitative and stratified aspects.

The subgroup analysis revealed several noteworthy trends. The patients with English as a second language (ESL) demonstrated a greater absolute improvement in understanding of their health conditions—from 22% pre-intervention to 76% post-intervention (a 54% increase)—compared to native English speakers, who improved from 34% to 71% (a 37% increase). Similarly, the patients who had completed only primary or secondary education showed a 55% improvement in self-management confidence (from 28% to 83%), compared to a 34% increase among those with higher education levels (from 38% to 72%). The gender-based differences were more modest but still evident: the female patients reported a 38% increase in willingness to adopt preventive behaviours (from 44% to 82%) compared to a 28% increase in the male patients (from 52% to 80%). These findings suggest that the intervention had the strongest relative impact on those with lower baseline health literacy and engagement.

### 3.5. Thematic Analysis of Student and Patient Narratives

The qualitative feedback from the student reflective work uncovered the central themes of empathy, simplification of medication terminology, and cultural adaptation of medication information. Several of the students reported that the experience had transformed their communication approach (see Table 4). One said, “I was surprised by the number of patients not understanding their medication. This altered the way I explain everything now”.

**Figure 4:**
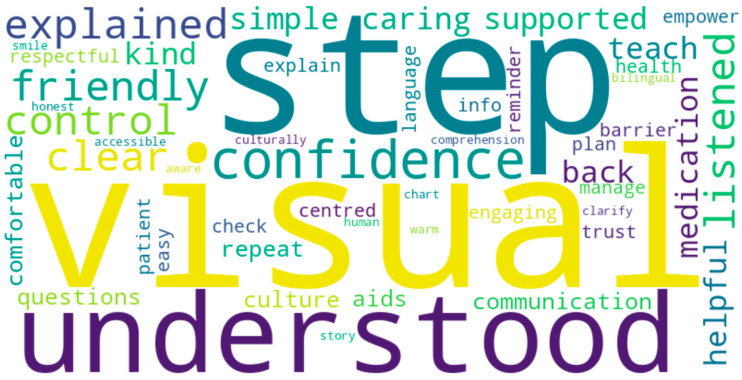
Common themes in patient feedback from education sessions. This word cloud highlights frequently mentioned words and phrases in patients’ free-text responses following the CBE intervention. Key themes include “confidence”, “understood”, “explained”, “friendly”, “trust”, and “clear”, suggesting that the sessions improved comprehension, comfort, and patient engagement. Terms such as “culturally aware”, “visual aids”, and “teach-back” indicate the perceived usefulness of interactive, inclusive communication strategies. The prominence of emotionally positive descriptors underscores the value patients placed on being listened to and respected.

The patient feedback ratified these findings. The patients valued the personalisation and explanation of the sessions (Table 5, Figure 4). One patient commented, “The student offered me advice that truly pertained to my situation”. Another said, “I felt for the first time like somebody explained something in a manner that was respectful of my background”.

## 4. Discussion

### 4.1. Key Results

This programme illustrated that integrating patient education into clinician activities through the application of CBE can significantly enhance both learning outcomes for students and patient health literacy. The results indicate that the students became more confident in simplifying medical concepts, using visual aids, and practicing culturally sensitive communication. The patients themselves reported greater health condition understanding, improved self-confidence in managing their conditions, and greater willingness to perform preventive health behaviours. Not only were these outcomes statistically significant but were accompanied by large effect sizes, perhaps indicating a strong educational and clinical effect.

The findings agree with the existing literature on experiential learning and service training models. Clinical experience in underserved populations has been reported through studies to promote communication skills, cultural competence, and professional identity development for medical students [20,21,22,23,24]. Through the qualitative findings in this study, the students gained a greater realisation of the value of health literacy and the social determinants of health, influencing their professional goals and patient care approach in the future [25,26].

For the patients, the CBE intervention closed the gaps in health literacy that have previously been shown to drive poor outcomes among underserved populations [3,6]. The patients said they felt more engaged, empowered, and ready to manage their chronic conditions. This is consistent with prior evidence showing that effectively delivered health education increases patient satisfaction, adherence, and involvement in healthcare decision-making [27,28,29,30,31]. The student feedback reflected that the preparation and giving of health education developed active listening, empathy, and social accountability. Holistic clinical care requires these relationship and reflective skills, and they are increasingly understood as key outcomes of contemporary medical education programmes [4,5,6,7,32,33,34].

### 4.2. Interpretation

These findings add to the evidence base for CBE as a transformative approach to medical education. They indicate that health education delivered by undergraduate medical students can be an effective way of narrowing health literacy disparities and of building key communication skills among forthcoming clinicians. Both objectives fit with the Ottawa Charter’s focus on empowering people and communities towards greater control over their health, and with calls for improved social accountability in healthcare education today [8,9,12].

These subgroup findings offer valuable insight into the differential impact of the intervention across patient demographics. The greater gains observed among patients with lower education levels and ESL backgrounds highlight the responsiveness of the programme to populations with higher health literacy needs. The modest differences between male and female patients also suggest a generally broad benefit, but with slightly stronger preventive engagement among women. From an educational perspective, these trends reinforce the importance of tailoring communication strategies and learning tools to the linguistic and cultural context of the patient. They also indicate that student-led community education can be particularly effective when focused on those who are typically underserved by traditional health communication strategies.

The success of the programme resides in its practical, two-way structure: the students learn useful skills, and patients gain useful insights into their health. This concurs with earlier research that illustrates the benefit of community partnerships and service learning in medical education [5,11,26]. The experiences of the healthcare providers also supported the relevance of the programme, with evidence of more aware and assertive patients in subsequent consultations.

This model also offers promise for expanded inclusion in community health interventions. Working with local healthcare providers, public health services, and community-based organisations, these types of CBE models may be scaled up and made sustainable beyond isolated placements [30,31,35]. Adding digital education tools, such as mobile applications, online video, or interactive portals, might enable patients to review and reinforce the content long after the student encounter has concluded [35,36].

The feasibility of implementing this model in other contexts—such as rural communities or international healthcare systems—depends largely on the availability of structured clinical placements, supervisory capacity, and community engagement. However, its core components—brief preparatory training, integration into routine care, and focus on underserved populations—make it highly adaptable. In rural or low-resource settings, collaboration with primary care clinics, use of tele-education platforms, and involvement of local community health workers could support implementation. To scale the model effectively, essential resources would include digital or modularised student training materials, standardised educational content, and mechanisms for continuity across student rotations. The simplicity and dual-benefit nature of the intervention render it a practical strategy for global adaptation within socially accountable medical education frameworks.

Another strength of the programme was that it was culturally responsive. Multilingual participants added diversity to the medical school population, making the sessions more culturally appropriate. The participants with low English language proficiency reacted especially enthusiastically, and the patient and student narratives underscored the value of cultural adaptation in the development of trust, understanding, and adherence [32,33,34]. The implications of these findings argue further for medical programmes to infuse cultural competency education across all educational levels.

Last but not least, the patient’s active participation in learning also became an essential theme. Visual aids and teach-back methods enabled collaborative learning, with the patients questioning, confirming understanding, and assuming control of their care plans. This fits with modern concepts of patient-centred care and shared decision-making that have been linked with improved outcomes and satisfaction [34,37].

### 4.3. Generalisability

While based on research in North West London, the results of this work have broad implications for other healthcare economies, especially those that cater to socioeconomically and/or linguistically marginalised populations. There are already established community-based models of medical education, and the extension of such frameworks into health literacy improvement seems a reasonable and effective next stage [1,2].

The findings indicate that a straightforward, replicable intervention—preparatory training and supervision of student-delivered education—can have notable benefits for patients and learners. The model may be applied in urban, rural, and global environments with flexibility for adaptation according to local health priorities and infrastructure.

This model of integrated community-based education holds promise for broader application across the health professions. While this study focused on final-year medical students, the structured nature of the intervention suggests it could be feasibly adapted for earlier-year students, nursing trainees, pharmacy students, or allied health learners [22,29,36]. Introducing these activities earlier in the curriculum may strengthen communication, empathy, and health promotion skills at a formative stage. It also supports interprofessional learning aligned with workforce realities and collaborative care.

Beyond the outcomes studied here, such interventions may influence student attitudes toward social accountability, career preferences for underserved settings, and patient safety awareness. Although this study did not systematically collect faculty perspectives, informal feedback from supervisors suggested improved student–patient engagement and greater continuity of care. Future work should include structured faculty input to evaluate the feasibility, supervision burden, and impact on teaching practice.

Integrating education for health literacy into formal educational curricula and clinical evaluation, and into mechanisms for regular collaboration with community partners, will facilitate its implementation on a broad scale. By doing so, institutions not only enhance medical education but help narrow health disparities. The work presented herein provides a model for moving medical education past the level of individual competence into making meaningful contributions in population health [36,38,39].

### 4.4. Limitations

In spite of the strength of this programme, there are a number of limitations. Firstly, as this was a pilot study focused on feasibility and within-subject change, a control group was not included. The findings aim to inform the design of future larger-scale controlled trials and so changes observed in this study cannot be attributed solely to the intervention. Although there were strong improvements observed in comparisons before and after the intervention, these findings must be treated with caution until independently verified by larger studies employing randomised or controlled designs [37,40,41]. Secondly, although the sample size for a pilot study was acceptable, it remains comparatively small. Specifically, the patient subgroup analysis by age, language, or chronic disease category was not possible, limiting our findings by granularity [40,42]. Thirdly, objective clinical outcomes such as blood pressure or healthcare utilisation were not assessed in this study, but future research should consider integrating these indicators to evaluate the longer-term health impacts of the intervention.

A limitation of this approach was the collection of self-reported measures using surveys and reflective journaling. Although these techniques elicited in-depth responses, they are prone to bias, such as social desirability and recall bias [43]. No objective outcome measures, such as tested health literacy tools, subsequent health care utilisation, or clinical indicators, were collected but should be included in subsequent studies. The short span of the intervention also limited the assessment of long-term behaviour change and health outcomes. A sustained effect must be measured through longitudinal designs and embedding in ongoing care pathways [43].

Validated health literacy tools such as TOFHLA or REALM were not employed in this study due to concerns about literacy burden and the need for brevity in a clinical setting. Instead, simplified pre–post questionnaires were used to maximise inclusivity. However, future studies should consider incorporating validated instruments to enhance objectivity and allow for standardised comparisons across settings.

Ultimately, the dual identity of both teacher and learner for the students created challenges. Some expressed difficulty juggling learning responsibilities with the necessity to be clinically accurate and efficient with time. This underlines the necessity of preparatory training, mentorship, and unambiguous expectations of roles within CBE environments for both patient and student support [40].

## 5. Conclusions

This research illustrates that not only do CBE programmes have the ability to build student skills but also that they have the capacity to measurably enhance health literacy among underserved patient groups. Through the inclusion of structured patient education within placement, the medical students were able to enhance their communication skills while, in turn, enabling the patients to gain greater knowledge and manage their health.

The evidence warrants a transformation in the way that CBE is envisioned—away from a mainly student-centred experience and towards a dual-impact model that also focuses on patient outcomes. With proper preparatory training, supervision, and partnering with local health systems, such interventions are scalable, sustainable, and achievable. It is a potent way of bringing medical education and public health goals into alignment, particularly in communities with persistent healthcare disparities.

As medical schools strive to carry out their mandate for social accountability, programmes such as ours provide a model for blending genuine community service with core educational objectives. Subsequent work should investigate how to scale up the approach through multidisciplinary collaborations, online education platforms, and longitudinal evaluation of the sustained impact.

## Figures and Tables

**Table 1:** Structured phases of the community-based education programme (description of the preparation, intervention, and evaluation phases, including key activities, learning objectives, and outcomes measured).

Phase	Key Activities	Learning Objectives	Outcomes Measured
Phase 1: Preparation	Two-day workshop on health literacy, communication, and cultural sensitivity. Training in simplifying language, using visual aids, and teach-back method. Review anonymised patient profiles. Role-playing exercises.	Understand health literacy’s impact on underserved communities. Develop culturally sensitive communication skills. Build confidence in patient education.	Pre-programme survey on confidence and understanding. Observations during role plays.
Phase 2: Intervention	Students conduct 20–30 min educational sessions on chronic disease and preventive health. Use visual aids, simplified language, and teach-back method. Engage with 2–4 patients over 4 weeks.	Apply knowledge in clinical settings. Develop skills in chronic disease education. Engage and empower patients.	Patient understanding via pre- and post-session surveys. Student performance assessed by supervisors. Patient satisfaction and feedback.
Phase 3: Evaluation	Pre- and post-programme surveys for students on confidence, skills, and cultural competence. Reflective journals. Pre- and post-session patient surveys. Multidisciplinary feedback.	Reflect on challenges and successes. Identify strategies for sustaining health literacy initiatives. Evaluate programme impact on students and patients.	Changes in students’ knowledge, confidence, and skills. Patients’ health literacy and self-management confidence. Feedback from healthcare providers.

**Table 2:** Quantitative results of student knowledge and confidence pre- and post-programme (statistical comparison of students’ abilities and confidence in simplifying medical concepts, using visual aids, and cultural sensitivity).

Metric	Pre-Programme Mean ± SD	Post-Programme Mean ± SD	Change	Key Observations
Confidence in simplifying medical concepts	2.8 ± 0.7	4.4 ± 0.5	+1.6 (*p* < 0.01)	Significant improvement in explaining medical terms in patient-friendly language.
Confidence in using visual aids and teach-back	2.5 ± 0.8	4.2 ± 0.6	+1.7 (*p* < 0.01)	Proficiency in using tools to enhance patient understanding.
Understanding of health literacy principles	3.2 ± 0.6	4.6 ± 0.3	+1.4 (*p* < 0.001)	Increased awareness of health literacy’s role in patient outcomes.
Cultural sensitivity in communication	3.1 ± 0.7	4.5 ± 0.4	+1.4 (*p* < 0.01)	Improved ability to tailor information to cultural and social contexts.
Ability to engage patients in discussions	40%	87%	+47%	Significant increase in comfort and effectiveness in patient interactions.
Competence in communication skills	42%	85%	+43%	Improved clarity and empathy in patient interactions, as assessed by supervisors.
Students identifying education as essential	54%	92%	+38%	Greater appreciation for health education in clinical practice.
Confidence in managing patient expectations	37%	81%	+44%	Students felt better equipped to manage patient concerns and expectations.

**Table 3:** Quantitative results for patient health literacy and confidence pre- and post-sessions (analysis of patients’ understanding of health conditions, confidence in self-management, and satisfaction with educational sessions).

Metric	Pre-Session Mean ± SD	Post-Session Mean ± SD	Change	Key Observations
Understanding of health conditions	27%	74%	+47%	Improved patient comprehension of their health conditions.
Confidence in self-managing chronic conditions	31%	79%	+48%	Patients felt more empowered to manage symptoms and follow treatment.
Satisfaction with educational sessions	-	91%	-	A majority found sessions helpful, highlighting the value of clear, personalised communication.
Willingness to adopt preventive health practices	48%	82%	+34%	Increased motivation to follow preventive health measures.
Clarity of information provided	41%	87%	+46%	Patients valued simple explanations, visual aids, and interactive methods.
Engagement during educational sessions	45%	89%	+44%	Patients were more engaged and comfortable asking questions.
Awareness of community health resources	32%	78%	+46%	Greater awareness of local healthcare resources.

**Table 4:** Thematic analysis of reflective journals: students’ experiences and insights (key themes such as empathy, simplifying medical language, cultural sensitivity, and confidence in teaching derived from students’ reflections).

Theme	Description	Illustrative Quotes	Key Implications
Empathy and Connection	Students developed a deeper understanding of patient challenges and the importance of empathy.	“Hearing patients describe their daily struggles made me realise the value of listening.”	Empathy is essential for effective patient-centred communication.
Simplifying Medical Language	Students learned to translate complex medical terms into clear language.	“A simple explanation changed a patient’s confidence in managing their health.”	Clear communication improves patient comprehension and adherence.
Cultural Sensitivity	Students recognised the need to tailor education to cultural contexts.	“Explaining health concepts in culturally respectful ways built trust.”	Culturally sensitive communication fosters trust and patient engagement.
Confidence in Teaching	Students gained confidence in educating patients effectively.	“I feel confident now in breaking down complex ideas.”	Confidence in teaching is crucial, especially in underserved communities.
Recognising Health Literacy Gaps	Students became aware of the impact of limited health literacy.	“I was shocked by how many patients didn’t understand their medications.”	Awareness of health literacy gaps drives efforts to integrate patient education.
Team Collaboration	Students valued feedback from supervisors and peers.	“Working with peers and getting feedback helped me refine my techniques.”	Collaborative learning enhances patient education delivery.

**Table 5:** Thematic analysis of patient feedback: perspectives on educational sessions (key themes from patients’ feedback, including clarity of information, empowerment, personalised education, and cultural sensitivity).

Theme	Description	Illustrative Quotes	Key Implications
Clarity of Information	Patients appreciated clear, simple explanations.	“The student explained my condition in a way I could finally understand.”	Clear communication improves patient understanding and confidence.
Empowerment and Confidence	Patients felt more capable of managing their health.	“I now feel more confident checking my blood sugar levels.”	Empowerment enhances self-management and health outcomes.
Personalised Education	Patients valued tailored advice for their specific needs.	“They gave me advice that really applied to my situation.”	Personalised education increases engagement and relevance.
Cultural Sensitivity	Patients appreciated respect for their cultural beliefs.	“The student was patient and respected my views, which built trust.”	Culturally sensitive communication strengthens trust and relationships.
Increased Awareness	Patients became more aware of their conditions and resources.	“I didn’t know about some local services, but now I feel more informed.”	Raising awareness empowers patients to seek help and manage health.
Engagement in Health Management	Patients felt more involved in healthcare decisions.	“The student asked for my opinion and explained how I could take better care of myself.”	Active participation improves health ownership and treatment adherence.
Appreciation of Interaction	Patients valued the time and effort spent educating them.	“The student made sure I understood everything”.	Building rapport enhances the educational impact of clinical encounters.

## Data Availability

The datasets generated and/or analysed during the current quality improvement project are not publicly available due ethical reasons but are available from the corresponding author (W.J.) upon reasonable request.

## Supplementary Materials

The following supporting information can be downloaded at: https://www.mdpi.com/article/10.3390/clinpract15060097/s1, HRA Decision Outcome.

## Appendix A

- Student Evaluation Form

A. Pre-Training Survey

Objective: To assess students’ baseline knowledge, confidence, and attitudes toward delivering patient education in underserved communities.

Instructions: Please rate the following statements on a scale of 1 to 5, where: 1 = Strongly Disagree, 2 = Disagree, 3 = Neutral, 4 = Agree, 5 = Strongly Agree.

Statement
- I understand the concept of health literacy and its impact on patient outcomes.
- I feel confident in explaining medical concepts in simple, patient-friendly terms.
- I am familiar with using visual aids and the teach-back method to enhance patient understanding.
- I am comfortable addressing cultural and social factors that influence patient health literacy.
- I feel confident engaging patients in discussions about their health and self-management.

Open-Ended Questions:

1. What challenges do you anticipate when educating patients about their health?
2. What do you hope to gain from this program?
3. How would you describe your current approach to patient education?

B. Post-Training Survey

Objective: To evaluate changes in students’ knowledge, confidence, and attitudes after participating in the program.

Instructions: Please rate the following statements on a scale of 1 to 5, where: 1 = Strongly Disagree, 2 = Disagree, 3 = Neutral, 4 = Agree, 5 = Strongly Agree.

Statement
- I feel confident in explaining medical concepts in simple, patient-friendly terms.
- I am familiar with using visual aids and the teach-back method to enhance patient understanding.
- I am comfortable addressing cultural and social factors that influence patient health literacy.
- I feel confident engaging patients in discussions about their health and self-management.
- This program enhanced my understanding of the importance of health literacy in patient outcomes.

Open-Ended Questions:

1. What was the most valuable lesson you learned during the program?
2. How has your approach to patient education changed after this experience?
3. What challenges do you still face when engaging patients in discussions about their health?

- Patient Feedback Form

A. Pre-Session Survey

Objective: To assess patients’ baseline understanding of their health conditions and confidence in self-management.

Instructions: Please rate the following statements on a scale of 1 to 5, where: 1 = Strongly Disagree, 2 = Disagree, 3 = Neutral, 4 = Agree, 5 = Strongly Agree.

Statement
- I understand the key aspects of my health condition and its management.
- I feel confident in managing my health condition (e.g., monitoring symptoms, following treatment plans).
- I feel comfortable asking healthcare providers questions about my health.
- I know where to find information or resources to help manage my health condition.

Open-Ended Questions:

1. What aspects of your health condition do you feel you need more help understanding?
2. What concerns or questions do you have about managing your health?

B. Post-Session Survey

Objective: To evaluate the impact of the educational session on patients’ understanding and confidence.

Instructions: Please rate the following statements on a scale of 1 to 5, where: 1 = Strongly Disagree, 2 = Disagree, 3 = Neutral, 4 = Agree, 5 = Strongly Agree.

Statement
- I better understand the key aspects of my health condition and its management.
- I feel more confident in managing my health condition.
- The student explained my health condition clearly and used terms I could understand.
- The session addressed my specific questions and concerns.
- I feel more comfortable asking healthcare providers questions about my health after this session.

Open-Ended Questions:

1. What did you find most helpful about the session?
2. Is there anything you still feel unsure about regarding your health?
3. What advice would you give to improve these sessions for future patients?

Reflective Journal Prompts for Students

Objective: To encourage critical reflection on the program and its impact on students’ learning and patient outcomes.

1. Describe a patient interaction where you felt you successfully enhanced the patient’s understanding of their health. What strategies did you use?
2. Reflect on a challenging moment during a patient education session. How did you address it, and what did you learn?
3. How has this experience influenced your approach to patient-centred care and health literacy?
4. What role did cultural sensitivity play in your interactions, and how did you tailor your approach to meet the patient’s needs?
5. What strategies will you use to incorporate patient education into your future clinical practice?
